# Genomic and Transcriptomic Analyses Reveals ZNF124 as a Critical Regulator in Highly Aggressive Medulloblastomas

**DOI:** 10.3389/fcell.2021.634056

**Published:** 2021-02-18

**Authors:** Zaili Luo, Xinran Dong, Jianzhong Yu, Yong Xia, Kalen P. Berry, Rohit Rao, Lingli Xu, Ping Xue, Tong Chen, Yifeng Lin, Jiyang Yu, Guoying Huang, Hao Li, Wenhao Zhou, Q. Richard Lu

**Affiliations:** ^1^Key Laboratory of Birth Defects, Children’s Hospital, Institutes of Biomedical Sciences, Fudan University, Shanghai, China; ^2^Department of Neurosurgery, Sichuan Academy of Medical Sciences and Sichuan Provincial People’s Hospital, Chengdu, China; ^3^Brain Tumor Center, Division of Experimental Hematology and Cancer Biology, Cincinnati Children’s Hospital Medical Center, Cincinnati, OH, United States; ^4^Department of Computational Biology, St. Jude Children’s Research Hospital, Memphis, TN, United States

**Keywords:** medulloblastoma, Asian cohort, whole exome sequencing, RNA-seq, subgroup-specific regulator, ZNF124, racial and ethnic disparities

## Abstract

Medulloblastoma (MB) is the most common malignant pediatric brain tumor, however, the mechanisms underlying tumorigenesis in different MB subgroups remain incompletely understood. Although previous studies of MB predisposition have been conducted in tertiary referral centers primarily in Caucasian cohorts, it is not unclear clear whether there exist population-specific genetic alterations in MBs. In this study, we investigated the contribution of genomic and transcriptomic alterations to the risk of malignant MB in the Chinese population (designated as the Asian cohort). We analyze the genomic and transcriptomic alterations of the Asian MB cohort by using a combination of whole-exome sequencing (WES) and RNA-deep-sequencing. In addition, we integrate publicly available data with the Asian MB cohort and identify a subset of potential MB-driving genes specifically enriched in each of the MB subgroups. We further characterize a newly identified group-3-enriched transcriptional regulator, ZNF124, and demonstrate that ZNF124 is critical for the growth of the most aggressive group-3 MB cells. Together, our analyses indicate conserved yet distinct genetic alterations and gene expression patterns of MBs between different ethnic groups. Our studies further provide an important resource for identifying potential tumor-driving factors in MBs, enhancing our understanding of the disease process for developing ethnically targeted therapies in patients with MB.

## Introduction

Medulloblastoma (MB) is the most common pediatric malignant brain tumor, accounting for approximately 63% of pediatric intracranial embryonal tumors and is associated with a high mortality and morbidity rates (Northcott et al., 2019). Patients receiving conventional multimodal therapy suffer from chronic sequelae such as physical disability and neurodevelopmental disorders for years after treatment completion, indicating a need for identification of effective targets for therapy (Narayan et al., 2019; De Medeiros et al., 2020). MBs originate within the developing cerebellum or brainstem (Waszak et al., 2018) though the etiology of MB tumorigenesis is not fully understood.

Based on distinct transcriptomic and epigenomic profiling, MBs can be classified into four broad subgroups: WNT, sonic-hedgehog (SHH), group 3 (G3) and group 4 (G4). Although a cohort of MBs arises from patients with risk-enhancing familial cancer syndromes such as Gorlin syndrome, Li-Fraumeni syndrome, and Turcot syndrome (Northcott et al., 2011), the majority of MBs arise due to somatic alterations in the genome. The WNT and SHH subgroups are usually associated with genetic alterations in WNT and SHH signaling, respectively. The WNT subtype comprises 10% of all MB patients and tends to have a favorable prognosis. The majority (97%) of WNT MBs are characterized by somatic mutations in *CTNNB1* or *APC* (Waszak et al., 2018), though somatic mutations in *DDX3X*, *SMARCA4*, and monosomy 6 are also detected in WNT MB (Northcott et al., 2012a). The SHH subgroup is prevalent in infants and adult patients, accounting for ∼25% of MBs (Srinivasan et al., 2016) and shows high heterogeneity in genetic changes and clinical presentation (Kumar et al., 2019). The typical genetic events that occur in the SHH signaling pathway are loss-of-function mutations or deletions including *PTCH1* (Smith et al., 2014), *GNAS* (He et al., 2014), and *GPR161* (Begemann et al., 2020), activating mutations in *SMO* (Twigg et al., 2016) and amplifications of *GLI1*, *GLI2*, and *MYCN* (Cavalli et al., 2017). Mutant *TP53* is another key hallmark in SHH MB, especially in the SHHa-MB subgroup (Louis et al., 2016).

The criteria for G3 and G4 subgroup classification are still controversial due to the lack of validated molecular driver events (less than 10% have a clear driver mutation) (Hovestadt et al., 2020). A recent reclassification of MBs has proposed that G3 and 4 tumors exist on a continuum between the previously described G3 and G4 molecular states (Northcott et al., 2017; Sharma et al., 2019). G3 MB is mainly characterized by *MYC* and *OTX2* amplifications or activation of *GFI1* and *GFI1B* in some cases (Northcott et al., 2017). A set of G4 MB is associated with enhancer-hijacking-mediated *PRDM6* overexpression (Northcott et al., 2014, 2017). Loss of 17p and gain of 17q (isochromosome 17q) is also prevalent in G3 and G4 MB (Northcott et al., 2017).

Most omics studies of MBs to date are from predominantly Caucasian cohorts with the genetic alterations and genomic features from Asian cohorts remaining poorly defined. It is not completely understood whether there are differences in genetic drivers of tumors between different racial ethnicities. Only two studies of MBs have been reported from Asian patients including a whole exome study of recurrent MB from only 17 pediatric patients in South Korea (Phi et al., 2018) and a small SHH-subtype MB cohort from Taiwan (Wu et al., 2020), however, a comprehensive analysis of Asian MB subgroups has not been conducted. In this study, we report the genomic landscape across a cohort of 89 MBs in the Han population from China. We further integrate molecular profiling of the new cohort of pediatric brain tumors with publicly available MB cohorts and found a set of novel potential driver genes in the individual MB subgroups, pointing to potential therapeutic targets. This dataset will serve as a foundation to gain greater fundamental insight into MB etiology and tumorigenic mechanisms for devising potential targeted therapies.

## Materials and Methods

### Study Subjects and Ethics Statement for Medulloblastoma Cohort

Human tumor and matched blood samples for WES or RNA-seq analysis were obtained with informed consent. Pediatric medulloblastoma tissues (89) and matched blood samples (57 used as controls) were collected from patients from the Children’s Hospital of Fudan University and the West China Hospital. All tumor tissues and blood were examined by whole exome sequencing (WES). Fifty-nine MB tissues were subject to RNA-sequencing (RNA-seq). Four normal apparent brain tissue samples from patients diagnosed with brain diseases other than brain tumor were used as a control for RNA-seq or WES. The study was approved by the Institutional Review Boards at the Children’s Hospital of Fudan University and West China Hospitals. The sequencing platform for WES and RNA-seq was an Illumina HiSeq X10, using pair end with 150bp for RNA-seq. The subtype for MB samples is identified by a set of subtype-specific marker gene expression: *WIF1*, *TNC*, *GAD1*, *DKK2*, and *EMX2* for WNT; *PDLIM3*, *EYA1*, *HHIP*, *ATOH1*, and *SFRP1* for SHH; *IMPG2*, *GABRA5*, *EGFL11*, *NRL*, *MAB21L2*, *NPR3*, and *MYC* for G3 and *KCNA1*, *EOMES*, *KHDRBS2*, *RBM24*, and *UNC5D* for G4.

### Whole Exome Sequencing and Somatic Variant Calling

Somatic variant analyses was conducted using the Genome Analysis Tool Kit (GATK) with best practices guidelines for genetic data preprocessing and variant calling (Mckenna et al., 2010; Mclean et al., 2019). Briefly, the fastq data files from WES were mapped to human genome (hg38) by the Burrows-Wheeler Aligner (BWA) (Li and Durbin, 2009) in the GATK4 module (Mckenna et al., 2010). We used HaplotypeCaller to call the germline mutations of single nucleotide variants (SNV) and insertion deletions (Indel) and followed the suggested pipeline of Mutect2 (Benjamin et al., 2019) to call the somatic SNVs and Indels. All variants were then annotated by ANNOVAR (Wang et al., 2010). Somatic mutations were filtered by the following criteria: (1) remove variants with alternative count reads smaller than 3; (2) remove allele frequency smaller than 5%; (3) remove variants with minor allele frequency larger than 0.01% in any of the public databases (ExAC, 1000 genome, ESP, Kaviar, HRC); (4) only include variants with ExonicFunc type in “frameshift deletion,” “frameshift insertion,” “non-synonymous SNV,” “stopgain,” and “stoploss.”

### Copy Number Variation (CNV) Analysis

The mapped bam files from WES were used for CNV analysis. We followed the somatic copy number variation pipeline from GATK4 CNV^1^. The final segment ratio files with CNV type annotation for all tumor samples were further annotated by AnnotSV (Geoffroy et al., 2018). Considering the limitation of WES in calling CNV, we only reported large CNVs. Somatic CNVs were filtered by the following criteria: (1) AnnotSV ranking smaller than 4; (2) segment log ratio smaller than 0.5; (3) CNV length smaller than 75% of its located chromosome p/q length.

### RNA-Seq Expression and Gene Fusion Analysis

The fastq data files from RNA-seq were processed by eXpress, a software package for efficient probabilistic assignment of ambiguously mapping sequenced fragments (Roberts and Pachter, 2013) with reference gene annotation from Gencode v29 (Harrow et al., 2012). The datasets were imported into DESeq2 (Anders and Huber, 2010) to get normalized expression matrix and differentiated expression statistics. We followed the pipeline from Arriba^2^ for the detection of gene fusions from RNA-seq data. Fusion events were filtered as follows: (1) remove events with confidence level at “low”; (2) remove events with site at intergenic regions; (3) remove events of the fused genes that only exist once in all samples; (4) remove events with both side gene type in “protein_coding,” “antisense,” “lincRNA,” “transcribed_unprocessed_pseudogene,” “transcribed_processed_pseudogene,” and at least one side with gene type in “protein_coding” and “lincRNA”; (5) remove events with only one read at the split point of the fusion gene; (6) remove events with the breakpoint in the 5′UTR or 3′UTR.

### Gene Annotation

The functional annotation of genes was collected from MSigDB^3^. The MB-related gene lists were collected from (1) marker genes; (2) DisGeNEt (Pinero et al., 2017); (3) 51 driver gene list from published datasets (Northcott et al., 2017); (4) literature search by easyPubMed (R package).

### Lentivirus shRNA Production

shRNAs against ZNF124 were designed based on the algorithm^4^ and the shRNA target sequences (shZNF124-1: GCAAGGACATATAAAGGCTCA and shZNF124-2: GCCAGTTCCCTTCAGAAACAC) were inserted to clone into pGreen-puro vector (SBI System Biosciences, Inc.). To produce lentiviruses, HEK293T cells were co-transfected with shRNA or GFP vector packaging using Lipofectamine 3000 reagent (Life Sciences). Supernatants were collected and filtered at 48 and 72 h following transfection. Viral supernatant was concentrated by centrifugation at 25,000 × rpm for 2 h at 4°C and used to infect cells (MOI = 5–10) overnight in the presence of 10 μg/mL polybrene. Cells were selected and maintained with puromycin (2 μg/ml). Gene expression was verified by real time PCR.

### Medulloblastoma Cell Culture and Proliferation Assays

Medulloblastoma cell line D458 was cultured in DMEM/F12 media with 10% FBS, 2 mM L-glutamine, and 1% Penicillin/Streptomycin at 37°C in an atmosphere of 5% CO_2_.

Cell proliferation was measured by WST-1 or BrdU assays. For WST-1 assay, we used the cell proliferation reagent WST-1 (Millipore Sigma) according to its protocol. For BrdU assay, procedures were performed according to the protocols as described^5^. Briefly, we added the BrdU (10 μM) into medium, cultured the cells for 3 h at 37°C in an atmosphere of 5% CO_2_, fixed cells with 4% paraformaldehyde and permeabilized with 0.3% Triton X-100. For BrdU staining, cells were treated with 1N HCl incubating 10 min on ice and neutralized by phosphate/citric acid buffer. BrdU antibody (Thermo Fisher Scientific, # MA3-071) was added into the cells and incubated overnight at 4°C. Mouse-anti BrdU (BD Bioscience, 1:500) antibody was used to label BrdU overnight at 4°C. DAPI counterstain was included in the final washes before the samples were mounted in Fluoromount G (SouthernBiotech) for microscopy. Cell images were quantified in a blinded manner. All immunofluorescence-labeled images were captured using a Nikon C2+ confocal microscope.

### Cell Apoptosis Analysis by Flow Cytometry

Medulloblastoma cells were washed with PBS and stained by Ghost Dye^TM^ UV450 (Tonbo Biosciences) for 30 min on ice to detect the viable and dying cells according to the protocol^6^. After staining, cells were washed with FACS buffer (ice-cold HBSS supplemented with 2% bovine serum) and washed twice with PBS before multiparameter flow cytometric detection on a BD LSRFortessa (Becton Dickinson, San Jose, CA, United States).

### Western Blotting and Quantitative PCR

Medulloblastoma cells were lysed in RIPA buffer, the protein concentration quantified (Bio-Rad), and the lysates were separated by 4–12% SDS-PAGE. Antibodies to ZNF124 (Abcam, ab168627) and GAPDH (Thermo Fisher Scientific, Catalog # 39-8600) were used in an incubation overnight. Bands were visualized with secondary antibodies conjugated to horseradish peroxidase (Bio-Rad) and ECL western blotting detection reagents (Pierce) per the manufacturer’s instructions.

Total RNA was extracted using the TRIzol Reagent (Invitrogen) and subsequently, 1 μg RNA was reverse-transcribed using the Advantages of a High-Capacity cDNA Reverse Transcription Kit (Bio-Rad). The cDNAs were then subject to real-time PCR analysis using Fast SYBR Green Master Mix in StepOnePlus Real-Time PCR system (Thermo Fisher Scientific). Primer sequences used were as follows:

*ZNF124* forward 5′- AGCCTTCCGTTACTCCAA; *ZNF124* reverse 5′- AGGGTTCTTCACCAGCAT; *SOX2* forward 5′- TGGGTTCGGTGGTCAAGT; *SOX2* reverse 5′- TCTGGTAGTGCTGGGACA; *P21* forward 5′- TCACTGTCT TGTACCCTTGTGC; *P21* reverse 5′- CTTCCTGTGGGC GGATTAG; *BAX* forward 5′- TTGCTTCAGGGTTTCATCCA; *BAX* reverse 5′- AGACACTCGCTCAGCTTCTTG; *PUMA* forward 5′- GTCCCCTGCCAGATTTGTGGC; *PUMA* reverse 5′- GACACTGCCGAGGGCACCAGG.

### Statistical Analysis

All analyses in this research were performed using Microsoft Excel or GraphPad Prism 6.00 (San Diego, CA, United States)^7^ or RStudio (R v.3.3.0, https://www.R-project.org/)^8^. Data are presented as means ± standard deviation (SD). Comparisons in gene expression among patients were evaluated by the Friedman non-parametric analysis of variance (GraphPad PRISM 8, GraphPad Software, United States). Differences were considered significant at *p* < 0.05. We used a Fisher’s exact test to assess the differences in mutation rates in each subgroup MB between tumor cohorts.

## Results

### Pediatric Brain Tumors in Asian Patients Have Similar Genetic Alterations to Their Caucasian Counterparts

**FIGURE 1:**
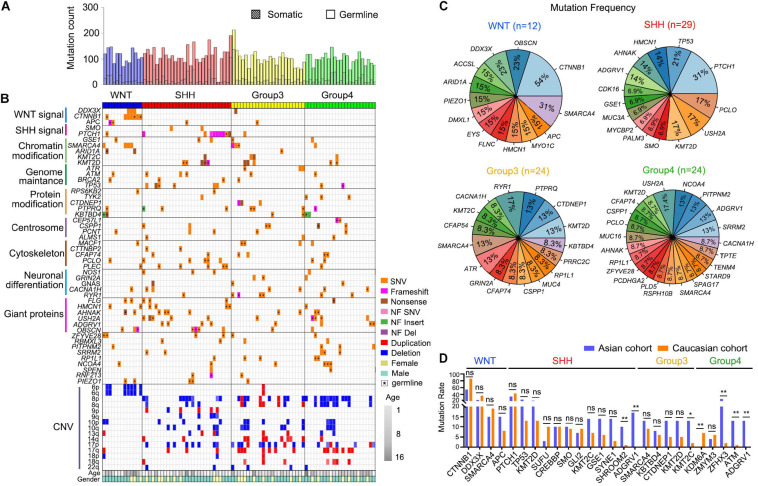
Genetic alteration landscape of Asian pediatric Medulloblastoma cohort. **(A)** Number of germline and somatic mutations in 89 pediatric medulloblastomas tested by WES. **(B)** OncoPrint summarizing recurrently altered genes and copy number variations of the 89 pediatric Medulloblastomas in the Asian cohort. **(C)** Recurrently mutated genes within the four subgroups of MBs. **(D)** Comparison between recurrently mutated genes in the Caucasian cohort (Northcott et al., 2017) based on WGS or WES data and the Asian cohort detected using WES data (this work). *P < 0.05, **P < 0.01, Fisher’s Exact test was used to assess the significance of the mutation rate.

We have analyzed a total of 89 MB patient tumor samples and 59 matched blood samples through whole exome sequencing (WES) and performed an analysis to determine the somatic and germline mutations via the Genome Analysis Tool Kit (GATK; Supplementary Table 1). We identified an average of 23.8 somatic mutations and 90.9 germline mutated genes on average for each Chinese pediatric brain tumor (Figure 1A and Supplementary Table 1).

Similar to the previously reported studies (Brennan et al., 2013; Hara et al., 2018), in WNT MBs we found frequent mutations in *CTNNB1* (54%, 7/13), *DDX3X* (23%, 3/13), *SMARCA4* (31%, 4/13), *ARID1A* (15%, 2/13), and chromosome 6p deletion (54%, 7/13) (Northcott et al., 2017). In SHH MBs, we found common mutations in *PTCH1* (31%, 9/29) and *TP53* (21%, 6/29). G3 MBs had recurrent mutations such as *KMT2D* (13%, 3/24), *PTPRQ* (13%, 3/24), and *CTDNEP1* (13%, 3/24) (Northcott et al., 2012b, 2017). G4 MBs exhibited recurrent mutations in *USH2A* (17.4%, 4/23), *ADGRV1* (13%, 3/23), *NCOA4* (13%, 3/23), and *PITPNM2* (13%, 3/23) (Figures 1B,C). We have further identified new sets of mutated genes associated with the centrosome (16%) and neural differentiation pathways (25%), especially in G3 (42%) and G4 (61%) MBs (Figure 1B). Interestingly, the genetic mutations in MBs did not exhibit apparent differences between genders except *TP53*, for which seven out of eight affected patients were female (Figure 1B and Supplementary Table 1).

When comparing the recurrent somatic mutations in the Asian cohort with the publicly available cohort (Northcott et al., 2017) with Caucasian populations, we found the mutation rates of most recurrently mutated genes are consistent between the two ethnic cohorts within MB subgroups (Figure 1D). However, there are some subtle differences. For instance, a recurrent *KDM6A* mutation, which is most frequently mutated in G4 MB in the Caucasian cohort (Northcott et al., 2017), but was not detected in G4 MBs in the Asian cohort (Figure 1D). The H3K27me3 demethylase KDM6A was reported to have a tumor suppressor function in medulloblastoma (Yi et al., 2020). Conversely, several recurrent mutations in the Asian cohort occurred rarely in the Caucasian cohort, including *SHROOM2*, encoding a key mediator of RhoA–ROCK pathway to inhibit tumor metastasis (Yuan et al., 2019), and *ADGRV1*, encoding an adhesion G protein-coupled receptor V1 associated with tumor invasion and metastasis (Aust et al., 2016), in SHH-MB, *KMT2C*, encoding a lysine methyltransferase 2C that regulates MB tumorigenesis (Roussel and Stripay, 2018) in G3-MB, *ZFHX3* (zinc finger homeobox 3), *ATM*, encoding ataxia telangiectasia mutated protein kinase, a DNA damage checkpoint-regulator, and *ADGRV1* in G4-MB (Figure 1D). These data suggest that heterogeneity exists in the prevalence of genetic mutations in MBs across distinct ethnic groups.

### Pediatric Asian MB Cohort Shows Transcriptomic Pathway Alterations Similar to Caucasian Patients

**FIGURE 2:**
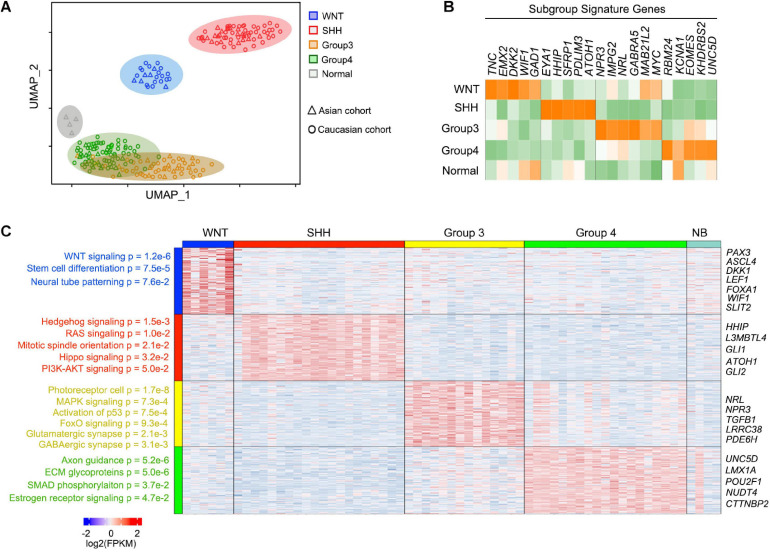
Signature genes for each MB subgroup and function enrichment. **(A)** UMAP plots for the 59 newly reported MBs and four normal cerebellar samples with similarity measured by the expression level of highly variable genes. The Caucasian MB cohort was used as the control of the known subgroup MB. **(B)** Heatmap of known marker genes for four subgroups of newly reported MBs showing significant differential expression across subgroups. **(C)** Heatmap and enriched pathway for the differential feature genes across MBs.

To characterize the gene expression pattern of the Asian MB cohorts, we performed RNA-seq for 63 MB tumors (Supplementary Table 2), including WNT (*n* = 6), SHH (*n* = 20), G3 (*n* = 14), G4 (*n* = 19) MBs and apparently normal brain controls (*n* = 4, marked as NB). Clustering analyses indicated that SHH and WNT formed distinct clusters, as opposed to G3 and G4 which are less well defined, consistent with previous subgrouping studies (Cavalli et al., 2017; Figure 2A). Previously established subgroup-specific marker genes clearly differentiate between each of these MB subgroups (Figure 2B). Signature genes of each MB subgroup from the Asian cohort were consistent with previously defined Caucasian MB subgroups (Figure 2C and Supplementary Table 3).

In the WNT MBs, highly enriched genes were related to WNT signaling pathways (*p* = 1.2e-6) including *DKK1*, *LEF1*, and *WIF1*, as well as stem cell differentiation regulators (*p* = 7.5e-5) such as *PAX3* and *FOXA1*. In SHH MBs, the enriched genes were associated with Hedgehog signaling (*p* = 1.5e-3), *RAS* (*p* = 1.0e-2), mitotic spindle orientation (*p* = 2.1e-2), as well as HIPPO (*p* = 3.2e-2), and PI3K-AKT (*p* = 5.0e-2) signaling. Although a cohort of patients in the G3 subgroup shared similar gene expression patterns with G4 MB patients overall, G3 MBs were distinguished by signatures of photoreceptor cells (*p* = 1.7e-8), or of MAPK (*p* = 7.3e-4) and FOXO (*p* = 9.4e-4) signaling, while signatures for axon guidance (*p* = 5.2e-6), ECM glycoproteins (*p* = 5.0e-6), SMAD phosphorylation (*p* = 3.7e-2), and estrogen receptor signaling (*p* = 4.7e-2) were enriched in G4 MBs (Figure 2C). The signature genes or pathways of each MB subgroup from the Asian cohort were consistent with previously defined MB subgroups (Figure 2C), suggesting that there is no major difference in MB subgroups among different racial populations based on gene expression patterns.

### Identification of Novel Gene Fusions in Asian Medulloblastomas

**FIGURE 3:**
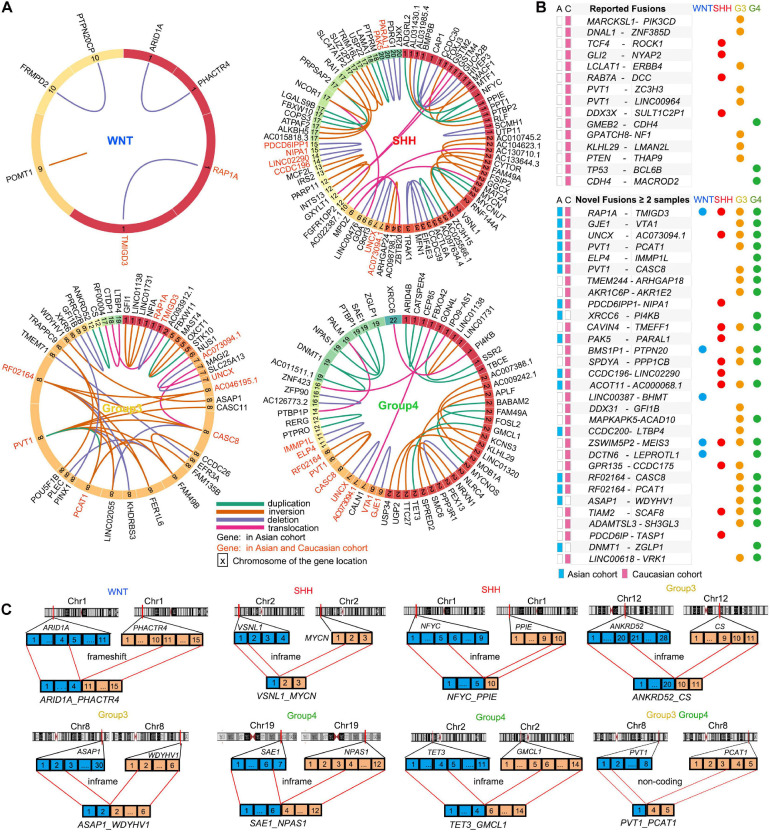
Gene fusion events in MB subtypes. **(A)** Gene fusions in WNT, SHH, Group3, and Group4 patients in the Asian cohort. The genes in red indicate the fusion genes also detected in the Caucasian cohort. The colors of the lines shows different types of fusions: duplication (green), inversion (brown), deletion (purple), and translocation (pink). The numbers in the block with different color indicate the numbered chromosomes where the gene located. **(B)** The reported fusion genes in the Caucasian cohort **(up)** and the novel fusion genes found in the Asian and Caucasian cohorts **(down)**. **(C)** Schematic summaries of high-confidence fusion transcripts targeting known or putative MB driver genes in the Asian cohort. Selected gene fusion events were shown: *ARID1A-PHACTR4* in WNT MB, *VSNL1-MYCN* and *NFYC-PPIE* in SHH MBs, *ANKRD52-CS, ASAP1-WDYHV1*, and *PVT1-PCAT1* in G3 MBs, *SAE1-NPAS1, TET3-GMCL1*, and *PVT1-PCAT1* in G4 MBs.

To identify the gene fusions within MB subgroups, we used the Arriba algorithm (Haas et al., 2019) to identify approximately 149 gene fusion events from 52 tumor samples including six WNT-MBs, 16 SHH-MB, 14 G3-MB, and 16 G4-MB in the Asian MB cohort (Figure 3A and Supplementary Table 4). Among the MB subgroups in the Asian cohort, we observed a greater number of fusion events in the SHH and G4 subgroups when compared to G3 and WNT subgroups. The most frequent fusion genes were *RAP1A*-*TMIGD3* in two WNT-MBs and one G3-MB, and *PVT1*-*CASC8* in two G3-MBs and one G4-MB, which is consistent with the Caucasian cohort (Northcott et al., 2017; Figure 3A). Notably, three fusion genes (*XRCC6*-*PI4KB, LINC01138-LINC01731*, and *DNMT1*-*ZGLP1*) were detected in at least two MB patients in the Asian cohort but not in the Caucasian cohort (Figure 3A). Conversely, a set of the reported MB fusion genes such as *MARCKSL1-PIK3CD, DNAL1-ZNF385D, TCF4-ROCK1, GLI2-NYAP2* in the Caucasian cohort were not detected in the Asian cohort (Figures 3A,B).

Our analysis also identified 31 novel fusion genes from at least two samples in the Asian and Caucasian MB cohorts (Figure 3B). In the Asian cohort, we identified MB subgroup-specific fusion genes such as a frameshift fusion gene *ARID1A-PHACTR4* in WNT-MB (Figures 3A,C), and in-frame fusion genes (e.g., *VSNL1-MYCN* and *NFYC-PPIE*) in SHH-MB (Figure 3C), in-frame fusion genes (e.g., *ANKRD52*-*CS* and *ASAP1*-*WDYHV1*) and frameshift gene fusions such as (e.g., *NF1A-GFI1* and *NUS1-SLC25A13*) in G3-MB (Figure 3C), as well as in-frame fusion genes (e.g., *DNMT1*-*ZGLP1*, *SAE1*-*NPAS1*, and *TET3*-*GMCL1*) and non-coding fusion genes (e.g., *DNMT1*-*AC011511.1*) in G4-MBs (Figure 3C and Supplementary Table 4). In addition, the fusion genes shared among different MB subgroups were also identified such as *GJE1*-*VTA1* (G3- and G4-MB), *PVT1*-*PCAT1* (G3- and G4-MB), and *UNCX*-*AC073094.1* (SHH-, G3-, and G4- MB; Figures 3B,C) in both Asian and Caucasian cohorts. The oncogenic effect of mutations in the fusion genes such as *ARID1A, PVT1, GFI1, MYCN, DNMT1*, and *TET3* has been identified in various tumors including medulloblastomas to regulate tumorigenesis (Bell et al., 2010; Northcott et al., 2014; Mathur, 2018; Mo et al., 2020; Onagoruwa et al., 2020; Wong, 2020), suggesting that the predicted inframe fusion proteins may possess oncogenic potential. Although the precise functions of the fusion genes in MB remain to be defined, our data suggest that novel gene fusion events might contribute to tumorigenesis in the MB cohorts.

### Identification of ZNF124 as a G3-MB-Enriched Gene

**FIGURE 4:**
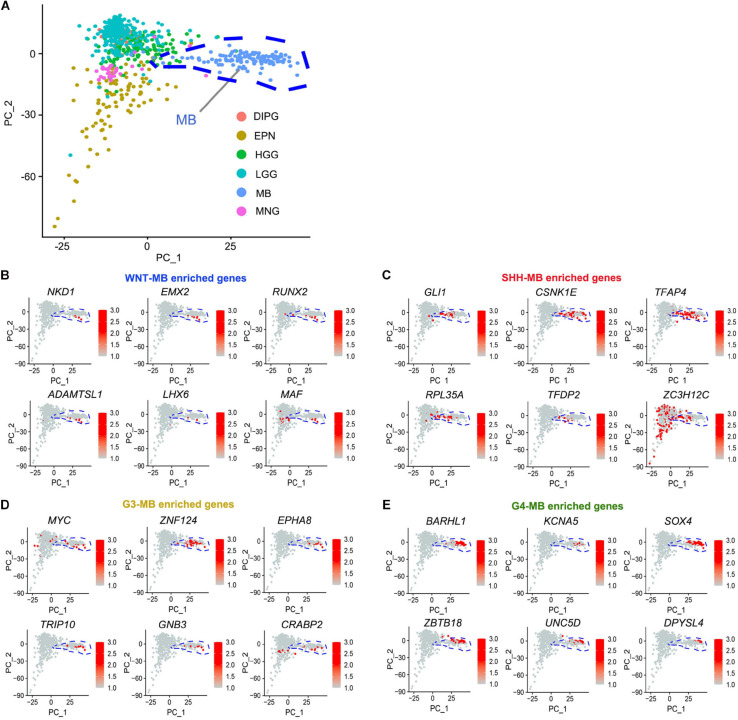
Identification of MB-subgroup-specific candidate driver genes. **(A)** Principal component analysis (PCA) of MB (133), EPN (98), DIPG (32), HGG (139), LGG (261), and MNG (34) based on the publicly available transcriptomics dataset (CBTTC, https://cbttc.org/). **(B–E)** The exclusive potential driver genes of WNT **(B)**, SHH **(C)**, G3 **(D)**, and G4 **(E)** MBs expression in brain tumors using CBTTC cohort.

To identify the candidate drivers specific to MBs, but not in other types of tumors, we integrated transcriptomic data from other pediatric brain tumors including ependymoma (EPN), high-grade glioma (HGG), low-grade glioma, (LGG), meningiomas (MNG), diffuse intrinsic pontine glioma (DIPG), and medulloblastomas (MB) from the CBTTC^9^ database and performed principal component analysis (PCA) to identify the MB-specific genes (Figure 4A). When integrating them with subgroup-enriched genes (Supplementary Table 4), we identified WNT signaling regulator *NKD1*, transcriptional regulators such as a RUNX family transcription factor (*RUNX2*), a homeobox-containing transcription factor (*EMX2*), and a LIM homeobox gene (*LHX6*) as potential WNT-MB subgroup-specific drivers (Figure 4B). In the SHH subgroup, we uncovered an effector of SHH signaling (*GLI1*), activating enhancer binding protein (*TFAP4*), and transcriptional regulator *TFDP2* as candidate SHH-MB subgroup-specific drivers (Figure 4C). In G3 MB, besides the previously known G3 driver MYC, we also identified candidate drivers including *ZNF124*, also known as *ZK7*, a transcriptional regulator linked to cell survival and tumorigenesis in other cellular contexts (Miyake et al., 2002), *TRIP10*, a member of the thyroid hormone receptor interactor family, and *CRABP2*, a member of the retinoic acid binding protein family (Figure 4D). In G4 MB, we identified several transcriptional regulators of neural development including *ZBTB18*, *BARHL1*, and *SOX4* as candidate driver genes in this subgroup (Figure 4E).

### Loss of Group 3 Specific Driver ZNF124 Attenuates Growth and Promotes Apoptosis of G3-MB Cells

**FIGURE 5:**
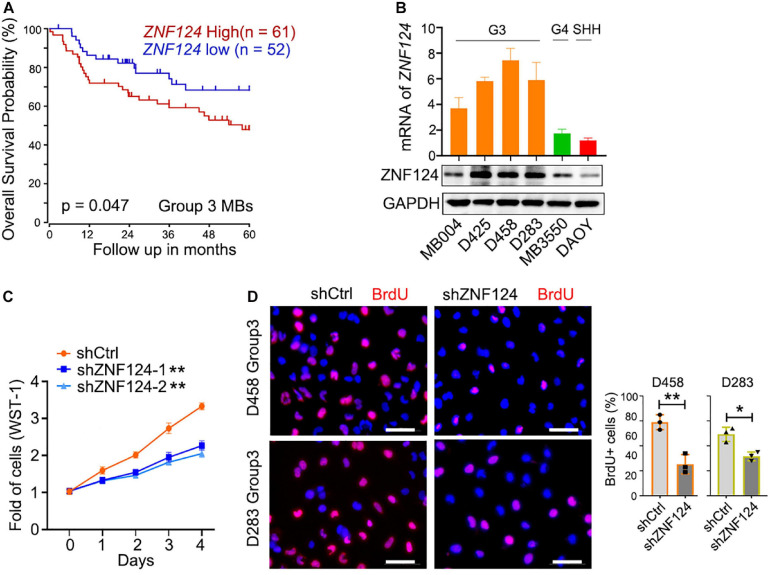
ZNF124 is specifically required for the growth of the aggressive G3MB cell. **(A)** Survival (5 years) analysis of the driver gene based on the ZNF124 expression using the publicly available datasets (GEO: gse85217). **(B)** RT-PCR experiment to detect ZNF124 mRNA **(upper)** and Western blotting to exam ZNF124 protein **(lower)** expression in human MB cell lines. Data represent means ± SD, *n* = 3 independent experiments. **(C)** Fold change in proliferation (as measured by WST-1) of control and shZNF124-expressing D458 cells. Values are means ± SD of five independent measurements across three replicate experiments. **p < 0.01, two-tailed unpaired *t*-test. **(D)** BrdU staining images for the control and shZNF124 D425 cells **(left)** and the quantification **(right)**. Data represent means ± SD, *n* = 3 independent experiments. *p < 0.05; **p < 0.01, two-tailed unpaired *t*-test.

**FIGURE 6:**
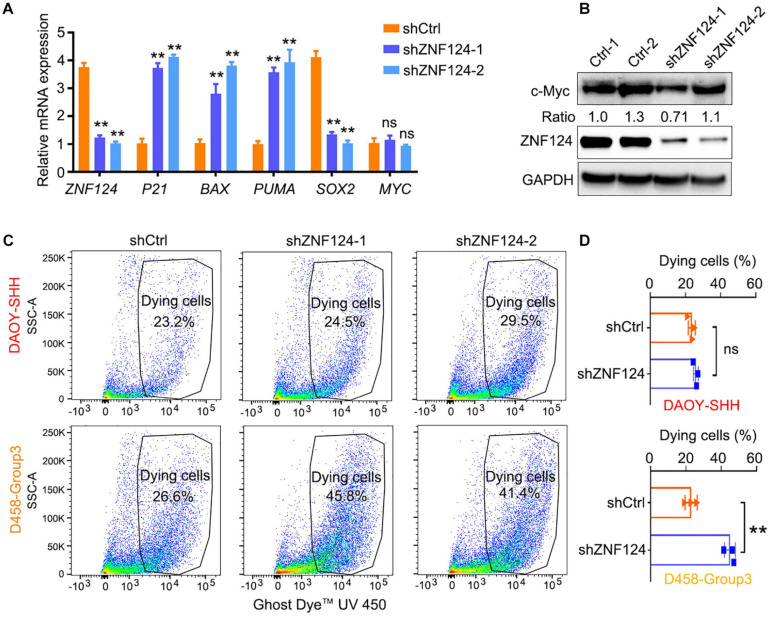
Knockdown ZNF124 promotes apoptosis of G3-MB cells. **(A)** RT-PCR data showed the efficiency of shZNF124 in MYC amplificated G3-MB D458 cells and the cell cycle inhibitor gene CDKN1A/P21, apoptosis genes BAX and PUMA and tumor stemness gene MYC and SOX2 mRNA expression. Data represent means ± SD, *n* = 3 independent experiments. **p < 0.01, two-tailed unpaired *t*-test. **(B)** Western blotting analysis of MYC and ZNF124 protein expression in human MB D458 cell after ZNF124 knockdown with Lenti-shRNA for 72 h. Ratio of MYC expression over GAPDH control was shown. **(C,D)** Dying cells were detected by flow cytometry in G3-D458 and SHH-DAOY cells after knockdown ZNF124 **(C)** and the comparison of the dead cells in control and shZNF124 treatment cells **(D)**. Data represent means ± SD, *n* = 3 independent experiments. **p < 0.01, two-tailed unpaired *t*-test.

We hypothesize that these MB-subgroup specific genes may play a role in MB tumorigenesis. To define and validate the function of the potential drivers in MB cell growth, we focused on ZNF124, a transcription factor whose loss has been linked to defects in cerebellar development (Poot et al., 2007), as a potential driver for G3-MB, the most aggressive G3 MB subgroup. High expression of *ZNF124* is associated with poor prognosis in G3-MBs (Figure 5A), suggesting that ZNF124 may have potential functions in G3 MB tumorigenesis. qPCR and western blotting analyses indicated that ZNF124 expression is higher in G3 MB cell lines (e.g., D425, D458, and D283) (Ivanov et al., 2016) compared with SHH-MB cells (DAOY) and G4 MB cells (MB3550; Figure 5B). Knockdown of *ZNF124* using lentiviral shRNA substantially reduced the proliferation of D458 cells, a G3 MB cell line (Ivanov et al., 2016), as measured by the cell proliferation assay (WST-1) and BrdU incorporation (Figures 5C,D). In addition, we found that lentiviral shRNA targeting *ZNF124* effectively inhibited expression of a stemness gene *SOX2* but significantly increased expression of a cell cycle inhibitor *P21/CDKN1A* and apoptosis-related genes such as *PUMA/BBC3* and *BAX* in G3 MB D458 cells (Figure 6A). However, ZNF124 knockdown in the MYC-amplified D458 G3-MB cells (Ivanov et al., 2016) did not significantly change the MYC expression at both mRNA and protein levels (Figures 6A,B), suggesting that ZNF124 does not directly regulate MYC expression.

Given that ZNF124 inhibits apoptotic death in other cell contexts (Kuramoto et al., 2000; Miyake et al., 2002), we then examined the proportion of apoptotic cells after ZNF124 knockdown in different MB cell lines. *ZNF124* knockdown increased cellular apoptosis in G3-MB D458 cells, but not SHH-DAOY cells (Figures 6C,D), suggesting that targeting *ZNF124* increases cell death specifically in G3-MB cells.

## Discussion

The characteristics of distinct MBs differ by their genetic alterations and gene expression profiles. An important aspect of MB tumorigenesis is its somatic mutations. However, the genetic characteristics of MBs in Asian populations are poorly understood. Our present analyses of the landscape of somatic mutations and transcriptomic profiles of MBs reveal similar genetic alterations and gene expression profiles between Caucasian and Asian cohorts, two large racial groups, suggesting that the major tumor driving events are conserved among different races.

In this study, most of the recurrent mutations in the Asian cohort are similar to Caucasian cohorts, however, there are novel gene fusion events unique to Asian MBs. The differences among racial or ethnic groups may lie in genetic, environmental, or behavioral factors that act alone or in combination. At present, whether and how the novel gene fusion events contribute to MB tumorigenesis or progression in each MB subgroup remain to be defined in Asian cohort. To our knowledge, our study provides the first comprehensive view of somatic mutations and gene expression profiles of MBs in Han Chinese population patients. Our study provides evidence of distinct genetic alteration events or heterogeneity among different racial and ethnic groups. Given that rare mutations can be responsible for severe effects in complex human disease and cancer (Mcclellan and King, 2010; Turajlic et al., 2019), it suggests the importance of tailoring an ethnicity-based genetic testing panel and treatment strategies for Asian patients. This Asian cohort of pediatric brain tumors presents an important resource for future studies that better represent this previously underrepresented population in pediatric brain tumor research.

Our genomic analysis of MBs identifies potential genetic alterations across different MB subtypes. In the Asian cohort and the publicly available MB cohorts, CNV is more prevalent in aggressive MBs (G3 and G4) when compared with WNT and SHH MBs and other pediatric brain tumors (Northcott et al., 2017). In addition, we identified unique inframe fusion proteins that may possess oncogenic potential in the Asian MBs, suggesting that the novel gene fusion events might contribute to tumorigenesis in the Asian MB cohort.

By performing a comprehensive analysis of this new Asian cohort in combination with publicly available cohorts, we identify a set of subgroup-specific potential driver genes across different MBs (Figure 4A). Particularly, we identified *ZNF124*, which is highly enriched in G3 MB, the most aggressive MB subgroup. High expression of *ZNF124* is associated with poor prognosis in MBs, suggesting that *ZNF124* may serve as a prognostic marker in G3 MB. ZNF124 has been shown to inhibit cell death in other cellular contexts (Kuramoto et al., 2000; Miyake et al., 2002). Interestingly, we show that *ZNF124* knockdown inhibits tumor cell growth and increases cell death specifically in G3-MB cells but not in SHH-MB cells, suggesting a unique role of ZNF124 in regulating the growth of the most aggressive G3 MB cells. Thus, our integrative transcriptomic profiling analysis for MB specific genes allows the identification of novel candidate driver genes that are critical for the growth of subgroup-specific MBs. Together, our present studies identify the genetic mutation landscape of the Asian ethnic population and provide an important reference for future genetic testing of cancer risk and potential targeted treatment in the patients with MBs.

## Data Availability Statement

The datasets presented in this study can be found in the NCBI Gene Expression Omnibus. The accession code is GSE164677.

## Ethics Statement

The study was approved by the Ethics Committee of Children’s Hospital of Fudan University, Shanghai, China, and conducted in accordance with the Declaration of Helsinki. Written informed consent to participate in this study was provided by the participants’ legal guardian/next of kin. Written informed consent was obtained from the individual(s), and minor(s)’ legal guardian/next of kin, for the publication of any potentially identifiable images or data included in this article.

## Author Contributions

QRL, ZL, and XD conceptualized, designed, and wrote the manuscript with input from all authors. ZL, LX, YL, JaY, and HL performed the experiments. XD and JyY performed data analysis. YX, PX, LX, GH, TC, HL, and WZ collected the samples. KB and RR revised the manuscript. All authors have read and approved the final manuscript.

## Conflict of Interest

The authors declare that the research was conducted in the absence of any commercial or financial relationships that could be construed as a potential conflict of interest.

## Footnotes

1 https://github.com/ding-lab/gatk4wxscnv

2 https://github.com/suhrig/arriba/

3 https://www.gsea-msigdb.org/gsea/msigdb/

4 https://rnaidesigner.thermofisher.com/rnaiexpress/

5 https://www.thermofisher.com/us/en/home/references/protocols/cell-and-tissue-analysis/protocols/brdu-labeling-and-detection-protocol.html

6 https://tonbobio.com/products/ghost-dye-uv-450

7 https://www.graphpad.com

8 https://www.rstudio.com/

9 https://cbttc.org/
